# Second International Electronic Conference on Medicinal Chemistry (ECMC-2)

**DOI:** 10.3390/ph10010020

**Published:** 2017-01-31

**Authors:** Annie Mayence, Jean Jacques Vanden Eynde

**Affiliations:** 1 Haute Ecole Provinciale de Hainaut Condorcet, 7330 Saint-Ghislain, Belgium; annie.mayence@condorcet.be; 2 Department of Organic Chemistry, University of Mons-UMONS, 7000 Mons, Belgium

**Keywords:** biological and therapeutic targets, biomolecules, drug development, drug discovery, medicinal chemistry, pharmacological evaluation

## Abstract

The second International Electronic Conference on Medicinal Chemistry, organized and sponsored by the publisher MDPI AG and the Journal *Pharmaceuticals*, took place in November 2016 on the SciForum website (www.sciforum.net/conference/ecmc-12). More than 150 authors from 22 countries participated in the event. Selected works presented during the scientific meeting are disclosed in this report.

## 1. Aim and Scope

The second International Electronic Conference on Medicinal Chemistry (www.sciforum.net/conference/ecmc-2) was organized by the journal *Pharmaceuticals*, published by MDPI AG. It took place on the Internet on 1–30 November 2016. The goal of the organizers was to invite researchers involved in the field of drug discovery and drug development to present their recent work to the scientific community and to share their results with academic and industrial groups from all over the world. The procedure selected for this second edition was constituted by four essential steps: (i) online submission of an abstract consisting of a brief description of the topic covered by the authors; (ii) evaluation of the scientific adequacy of the subject with the scope of the conference; (iii) online submission of a slide show or a poster; (iv) evaluation of the scientific and general qualities of the full submission. Forty communications were accepted and selected abstracts are disclosed hereafter.

## 2. Presentations

### 2.1. Interaction of Zinc(II) and Copper(II) Terpyridine Complexes with Biomolecules (A001)

SelimovićEnisaSoldatovicTanja*Department of Chemical-Technological Science, State University of Novi Pazar, Vuka Karadžiča bb, 36300 Novi Pazar, Serbia*Correspondence: tsoldatovic@np.ac.rs

Transition metal ions exhibit a unique role in diverse biological activities of proteins by acting as cofactors. In particular, zinc and copper ions modulate enzymes activities as well as many catalytic and oxidative/reductive processes (Bertini I., et al., *Biological Inorganic Chemistry. Structure and Reactivity*, University Science Books: Sausalito, CA, USA, 2007; Roat-Malone R.M. (Ed.), *Bioinorganic Chemistry: A Short Course*, John Wiley & Sons, Inc., Hoboken, NJ, USA, 2002).

The kinetics and mechanism of the substitution reactions of dichlorido [ZnCl_2_(terpy)] and [CuCl_2_(terpy)] (terpy = 2,2′:6′,2′′-terpyridine) with biologically relevant ligands have been studied as a function of nucleophile concentrations at pH 7.38, under pseudo-first-order condition by UV-Vis spectrophotometric techniques. The interactions of Cu(II) and Zn(II) complexes with tripeptide glutathione (GSH) were investigated under pseudo-first-order conditions with respect to the complex concentration. 

For the substitution process of Zn(II) complex with glutathione (GSH), pre-equilibrium and chelate formation have been noted. The [CuCl_2_(terpy)] is more reactive than [ZnCl_2_(terpy)] complex and the second-order rate constants for the first step follow the order of reactivity: GSH > DL-Asp > L-Met > 5’-GMP ~ 5’-IMP for Cu(II) complex, while for Zn(II) the order of reactivity is: DL-Asp > L-Met > GSH ~ 5’-GMP > 5’-IMP. The results are discussed in terms of mechanisms of interactions between metalloproteins and biomolecules.

**Acknowledgments:** The authors gratefully acknowledge financial support from State University of Novi Pazar, Novi Pazar, Republic Serbia and T. Soldatović also gratefully acknowledges financial support from Ministry of Education, Science and Technological Development, Republic of Serbia (Project No. 172011). 

### 2.2. Antibacterial Activity of Zinc(II) and Copper(II) Terpyridine Complexes (A002)

LičinaBraho^1^SelimovićEnisa^2^SoldatovićTanja^2^*1Department of Biomedical Science, State University of Novi Pazar, Novi Pazar, Vuka Karadžiča bb, 36300 Novi Pazar, Serbia2Department of Chemical-Technological Science, State University of Novi Pazar, Vuka Karadžiča bb, 36300 Novi Pazar, Serbia*Correspondence: tsoldatovic@np.ac.rs

Zinc(ll) and copper(II) complexes with organic molecules are used in clinical medicine, e.g., (i) a complex of zinc(ll) acetate with erythromycin is used for ache therapy; (ii) copper chelating agents were developed to treat Wilson disease, an autosomal recessive genetic disorder that causes copper accumulation primarily in the liver (Feucht, C.L., et al. *J. Am. Acad. Dermatol.* **1980**, *3*, 483–491; European Association for Study of Liver, *J. Hepatol*. **2012**, *56*, 671–685).

In general, organic ligands can contribute to better transport of metal ions through the lipophilic regions of cell membranes. However, it is also possible that some metal complexes are not able to reach their site of action in sufficient concentration due to their decreased solubility (R. B. Martin in *Metal lons in Biological Systems*, Vol. 20, H. Sigel, (Ed.) Marcel Dekker: New York, NY, USA and Basel, Switzerland, 1986).

The antibacterial activity of model [ZnCl_2_(terpy)] and [CuCl_2_(terpy)] complexes was tested against seven strains of bacteria. The complexes were more effective against Gram-positive than Gram-negative bacteria. Between complexes, stronger effect was observed for [CuCl_2_(terpy)] complex. The best effect was exhibited against *Sarcinalutea* (5 mg/mL). *Escherichia coli* showed low sensitivity to both complexes. Results of the study of the antibacterial activity suggest an absence of permeability of the complexes through the membrane proteins.

**Acknowledgments:** The authors gratefully acknowledge financial support from State University of Novi Pazar, Novi Pazar, Republic Serbia and T. Soldatović also gratefully acknowledges financial support from Ministry of Education, Science and Technological Development, Republic of Serbia (Project No. 172011). 

### 2.3. Bioprospecting of Asteraceae Medicinal Plants of Pakistan for their Associated Bioactive Endophytic Actinomycetes for New Drug Targets (A004)

TanvirRabia^1^,^2^,^3^*SajidImran^2^HasnainShahida^2^,^4^KulikAndreas^5^GrondStephanie^3^1University Diagnostic Lab (UDL), University of Veterinary and Animal Sciences (UVAS), 54000 Lahore, Punjab Pakistan2Department of Microbiology and Molecular Genetics, University of the Punjab, Quaid-e-Azam Campus, 54590 Lahore, Punjab, Pakistan3Institut für Organische Chemie, Auf Der Morgenstelle 18A, Eberhard Karls Universität Tübingen, 72076, Tübingen, Germany4Department of Microbiology and Molecular Genetics, University of the Punjab, Quaid-e-Azam Campus, 54590, Lahore, Punjab, Pakistan; The Women University of Multan, Punjab, Pakistan5Mikrobiologie/Biotechnologie, Interfakultäres Institut für Mikrobiologie und Infektionsmedizin, Eberhard Karls Universität Tübingen, Auf der Morgenstelle 28, 72076 Tübingen, Germany*Correspondence: rabia.tanvir@uvas.edu.pk

Since the beginning of mankind, plants have been used as the source of medicinal agents, thereby becoming a major course to discovering new drugs. The practice of using traditional medicine is prevalent in Pakistan, that has a rich history of herbal plants being used by Hakims in folk medicine (Unani medicine). The Asteraceae family is the largest plant family in Pakistan, with plants of considerable medicinal importance. Endophytes include all organisms that symptomlessly colonize the living internal tissues of their hosts during a variable period of their lifetime. There they produce a broad variety of bioactive secondary metabolites with unique structures that are advantageous for the plant. Endophytic actinomycetes also colonize the internal tissues of plants without causing any visible changes or damage. They exploit an unusual habitat and considering this, this may enable them to possess the potential to produce bioactive compounds similar to those of their host plant. Our study explores the bioprospecting potential related to endophytic actinomycetes of Asteraceae medicinal plants of Pakistan. After isolation and identification the endophytes were screened for their bioactive metabolites potential for new drug targets. This included extensive biological and chemical screening. The bioactive compounds were purified through column chromatography and final identification was done through HPLC-MS and NMR. The purified compounds were observed to be extremely potent with promising antimicrobial potential against major pathogens including algae and fungal strains as well as possessing antioxidant and cytotoxic potential.


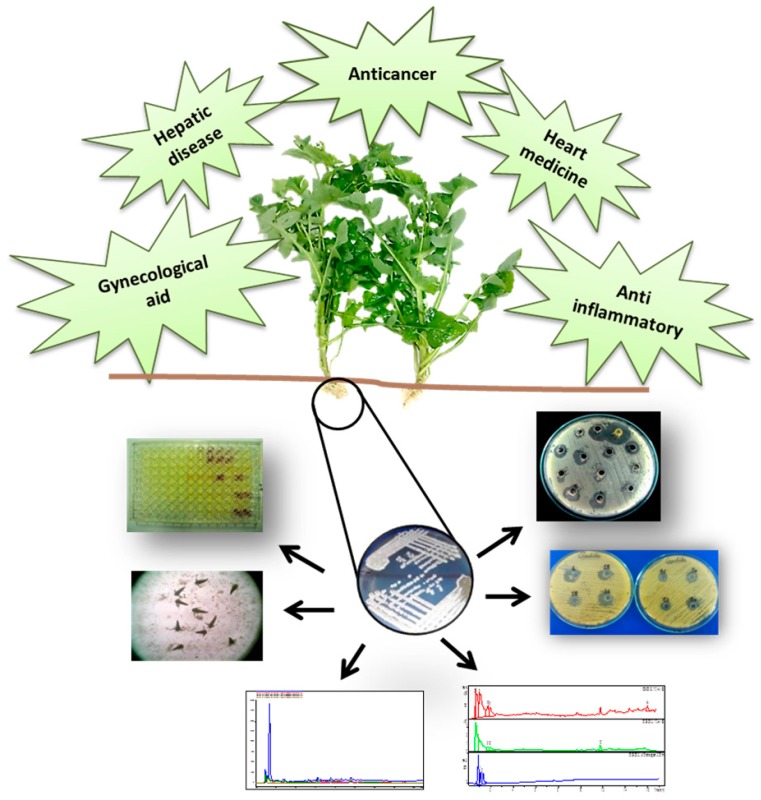


**Acknowledgments:** The Higher Education Commission (HEC) Pakistan is greatly acknowledged for the grant 1-/HEC/HRD/2012/2301 under the IRSIP program to the recipient Rabia Tanvir. Yi Zhang at the Guangdong Ocean University China provided the *E. coli* K-12 strain for which the authors are greatly thankful.

### 2.4. Artemisinin: Tentative Mechanism of Action and Resistance (A008)

TorrensFrancisco^1^*RedondoLucía^2^CastellanoGloria^2^1Institut Universitari de Ciència Molecular, Universitat de València, Edifici d’Instituts de Paterna, P. O. Box 22085, E-46071 València, Spain2Departamento de Ciencias Experimentales y Matemáticas, Facultad de Veterinaria y Ciencias Experimentales, Universidad Católica de Valencia *San Vicente Mártir*, E-46001 València, Spain*Correspondence: torrens@uv.es

Sesquiterpene lactones constitute a large class of secondary plant metabolites, which bear α-methylene-γ-lactone groups as common structural element and display a number of bioactivities (Castellano, G., et al. *Int. J. Mol. Sci*., submitted for publication). Every year, 1–2 million people living in the tropics and subtropics die of malaria, the most infectious disease in the world today. The lactone artemisinin and its derivatives are currently the most effective treatments vs. malaria. Artemisinins are derived from extracts of sweet wormwood (*Artemisia annua*) and are well established for the treatment of malaria, particularly highly drug-resistant strains. They resulted in one of the most significant advances in the treatment of malaria since the discovery and first use of quinine over 300 years ago. Their efficacy also extends to phylogenetically unrelated parasitic infections, e.g., schistosomiasis. They showed potent and broad anticancer properties in cell lines and animal models. What hope does this drug offer for the future? Artemisinin and its derivatives present structures that differ different from the classical quinoline and contribute to the mechanism of action, whereby the pharmacophoric –O_1_–O_2_– peroxide linkage in the endoperoxide ring literally *triggers* artemisinin to *explode*. Artemisinin is poorly soluble in water (and oil). When creating the first generation of artemisinin derivative drugs in 1970s, the overriding goal was to improve its solubility characteristics, so that artemisinin derivatives be more easily formulated and efficiently delivered. Despite much research, artemisinin remains the only known natural product to contain a 1,2,4-trioxane ring and various *Artemisia* species continue to be the only known natural sources. Phytochemical investigation of these species revealed an abnormally wide range of other endoperoxides and hydroperoxides, many of which were not tested for their antimalarial activity. In this communication the design, synthesis and cytotoxicity of some novel dihydroartemisinin-coumarin hybrids produced via click chemistry are described.


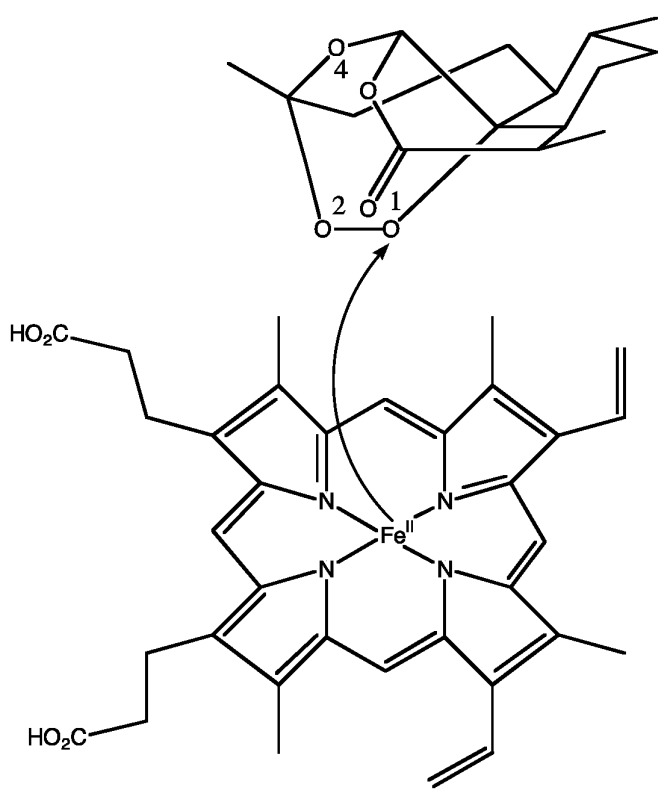


**Acknowledgments:** This work was supported from the Spanish Ministerio de Economía y Competitividad (Project No. BFU2013-41648-P), EU ERDF, Generalitat Valenciana (Project No. PROMETEO/2016/094) and Universidad Católica de Valencia *San Vicente Mártir* (Project No. PRUCV/2015/617).

### 2.5. Highly Conserved WNV Genomic RNA Domains Are Potential Targets of Antiviral RNA Aptamers (A009)

Fernández-MolinaCristinaRomero-LópezCristina*Berzal-HerranzAlfredo*Institute of Parasitología y Biomedicina “López-Neyra” (IPBLN-CSIC), Department of Molecular Biology, 18016 Armilla, Granada, Spain*Correspondence: cristina_romero@ipb.csic.es (C.R.-M.); aberzalh@ipb.csic.es (A.B.-H.)

West Nile virus (WNV) is an enveloped, single-stranded, positive RNA virus belonging to the *Flavivirus* genus (*Flaviviridae* family). Different WNV strains have been involved in important outbreaks of human and animal diseases, and WNV infection is now considered an emerging world health problem. Like other RNA viruses, WNV has a compact RNA genome that efficiently stores all the information required for the completion of the infectious cycle. The efficiency of this storage system is attributable to supracoding elements, i.e., discrete, structural units that play essential functions. The information coded in the form of structural elements overlaps and complements the protein coding information and is highly conserved across *Flavivirus* spp. These elements therefore offer interesting potential targets for novel therapeutic agents. We have applied a SELEX procedure to isolate RNA aptamers against the essential 3’ untranslated region (3’UTR) of the WNV genome. Aptamers are oligonucleotides that efficiently and specifically bind to a ligand molecule. Starting from a theoretical highly sequence-variable population consisting of more than 10^18^ different molecules, and after six rounds of selection we have identified two main groups of aptamers defined by conserved sequence motifs complementary to highly conserved counter parts within essential structural elements of the WNV genome. Current results point out the potential of these essential functional genomic RNA elements to efficiently bind RNA molecules, therefore to be involved in RNA-RNA interactions, offering a potential of being used as targets of antiviral agents based on nucleic acids. Further biochemical and functional studies are being performed to characterize the antiviral activity of identified RNA aptamers.

**Acknowledgments:** We thank Juan Carlos Sáiz for the WNV genomic clone, Beatriz Berzal-Herranz and Vicente Augustin for excellent technical assistance. This work was supported by the Spanish *Ministerio de Economía y Competitividad* [BFU2015-64359-P] to A.B.-H., which is partially supported by FEDER funds from the EU.

### 2.6. Synthesis of Bioactive 2-(Arylamino)thiazolo[5,4-f]-quinazolin-9-ones via the Hügershoff Reaction or Cu-Catalyzed Intramolecular C-S Bond Formation (A010)

HédouDamien^1^DubouilhCarole^1^LoaëcNadège^2^,^3^MeijerLaurent^3^FruitCorinne^1^BessonThierry^1^*1Normandie Univ, UNIROUEN, INSA Rouen, CNRS, COBRA, 76000 Rouen, France2Protein Phosphorylation & Human Disease group, Station Biologique, 29680 Roscoff, France3Manros Therapeutics, Centre de Perharidy, 29680 Roscoff, France*Correspondence: thierry.besson@univ-rouen.fr

The occurrence and properties of the thiazole ring in various natural and synthetic products have been the interest of many research groups on account of its useful biological properties. In this context, our research group is mainly invested in the synthesis of C,N,S-containing bioactive molecules able to modulate the activity of deregulated kinases (CDK5, GSK-3, CLK1, CK1 and the dual-specificity kinase DYRK1A) involved to some extent in Alzheimer’s disease (AD) studied (Logé, C., et al. *Eur. J. Med. Chem*. **2008**, *43*, 1469–1477; Testard, A., et al. *Bioorg. Med. Chem.* *Lett.* **2006**, *16,* 3419–3423; Foucourt, A., et al. *Molecules* **2014**, *19*, 15411–15439; Foucourt, A., et al. *Molecules* **2014**, *19*, 15546–15571).

A library of thirty eight novel thiazolo[5,4-f]quinazolin-9(8H)-one derivatives (series I, II, III and IV) was prepared via the Hügershoff reaction and a Cu catalyzed intramolecular C-S bond formation, helped by microwave-assisted technology when required. The efficient multistep synthesis of the key 6-amino-3-cyclopropylquinazolin-4(3*H*)-one has been reinvestigated and performed on a multigram scale from the starting 5-nitroanthranilic acid. 


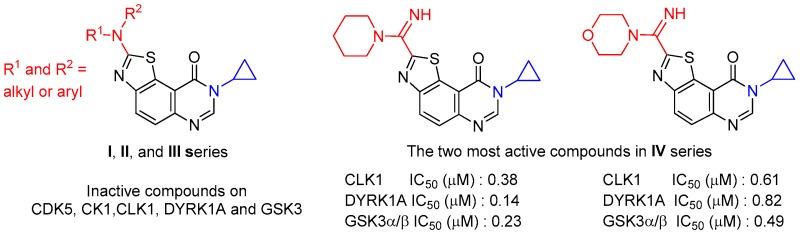


The inhibitory potency of the final products was evaluated against five kinases involved in Alzheimer’s disease and showed that some molecules of the IV series described in this communication are particularly promising for the development of novel multi-target inhibitors of kinases (Hédou, D., et al. *Molecules* **2015**, *21*, 794–799).

**Acknowledgments:** Financial support from the MESR (French Ministère de l’Enseignement Supérieur & de la Recherche) is gratefully acknowledged for the doctoral fellowships to D.H. We thank the LABEX SynOrg (ANR-11-LABX-0029) for financial support (J.G.). We also acknowledge Milestone S.r.l. (Italy) for provision of multi-mode microwave reactor (Start STM) and for technical support. This research was partly supported by grants from the ‘Fonds Unique Interministériel” (FUI) TRIAD (LM) projects, the “Fondation Jérôme Lejeune” (LM), and an FP7-KBBE-2012 grant (BlueGenics) to LM.

### 2.7. Antimicrobial Peptide Prodrugs and Mimetics (A011)

FordeÉanna^1^,^2^SchütteAndré^3^Molero-BondiaAndrea^1^SweeneyLouise^4^Mac LoughlinRonan^4^ReevesEmer^5^GreeneCatherine^5^HumphreysHilary^2^,^6^MallMarcus^3^Fitzgerald-HughesDeirdre^2^DevocelleMarc^1^*1Department of Pharmaceutical & Medicinal Chemistry, Royal College of Surgeons in Ireland, 123 St Stephen’s Green, Dublin 2, Ireland;2Department of Clinical Microbiology, Royal College of Surgeons in Ireland, Education & Research Centre, Beaumont Hospital, Beaumont, Dublin 9, Ireland3Department of Translational Pulmonology, Translational Lung Research Center Heidelberg (TLRC), University of Heidelberg, Heidelberg D-69120, Germany4Aerogen Ltd, Galway Business Park, Dangan, Galway, Ireland5Department of Medicine, Royal College of Surgeons in Ireland, Education & Research Centre, Beaumont Hospital, Beaumont, Dublin 9, Ireland6Department of Microbiology, Beaumont Hospital, Dublin 9, Ireland*Correspondence: mdevocelle@rcsi.ie

Antimicrobial Peptides (AMPs) represent one of the most durable and effective defences of multicellular organisms against bacterial infections. These cationic and amphipathic peptides represent promising leads for the development of antibiotics combatting the resistance of bacteria to antibiotics. However, their clinical applications have often been limited by an inadequate margin of safety (Zasloff, M. *Nature* **2002**, *415*, 389–395).

A prodrug approach can overcome a toxicity barrier in drug delivery. Prodrugs of AMPs can be generated by transiently reducing or annulling their net positive charges by attaching a negative promoiety through a linker which can be degraded by an enzyme (bacterial or human) confined to sites of infection. For example, neutrophil elastase (NE), a human protease involved in chronic airway inflammation and infections associated with cystic fibrosis (CF), can restore the cationic property of AMPs modified with oligo-glutamate promoieties. Consequently, their bactericidal activities against the CF pathogen P. aeruginosa are restored by NE in CF bronchoalveolar lavage fluids. The potential of this prodrug approach in reducing the safety barrier in the clinical use of AMPs has also been demonstrated in vivo, in a murine model of lung delivery (Forde, É., et al. *Antimicrob Agents Chemother*. **2016**, *60*, 2813–2821). Finally, an in vitro nebulisation study performed with a vibrating mesh nebuliser demonstrates that a high level of dosing in the lung can be achieved for this AMP prodrug.


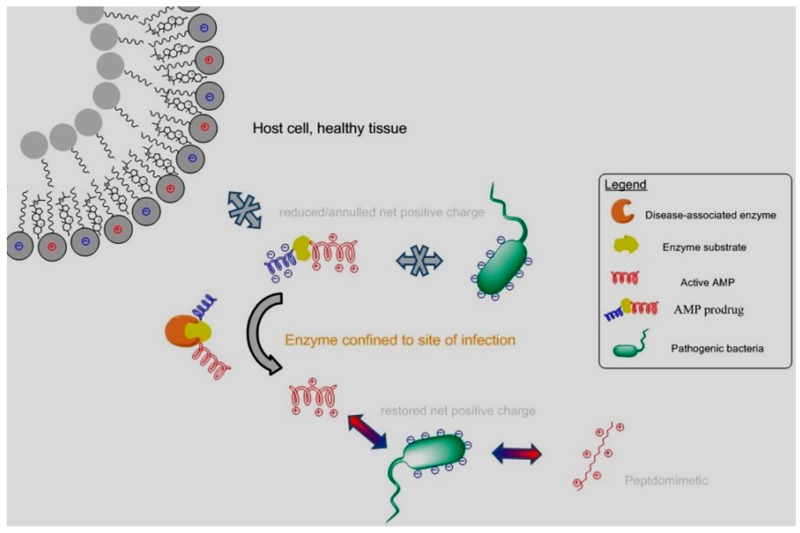


In parallel, a novel class of peptidomimetics with antimicrobial activities similar to AMPs, against Gram-positive bacteria, has been developed. Their spectrum of activity is currently extended to Gram-negative organisms.

**Acknowledgments:** This work was funded by the Higher Education Authority, Ireland, under the BioAT program in Cycle 5 of the Programme for Research in Third- Level Institutions and by the Science Foundation Ireland under grant numbers 06/RFP/CHO024/602 EC07 and 13/TIDA/B2657. Funding for a research visit to Heidelberg, Germany, was provided by a Microbiology Society research visit grant.

### 2.8. Development of New Aromatic Sulfonamides as Potential Antiglaucoma Agents (A012)

RemkoMilan^1^*GregáňFridrich^2^1Department of Pharmaceutical Chemistry, Faculty of Pharmacy, Comenius University in Bratislava, Odbojárov 10, 832 32 Bratislava, Slovakia2Department of Chemistry, Faculty of Natural Sciences, Matej Bel University, 974 01 Banská Bystrica, Slovakia*Correspondence: remko@fpharm.uniba.sk

Many sulfonamides with the general formula R-SO_2_NH_2_ constitute an important class of inhibitors of the zinc enzyme carbonic anhydrase (CA) used in antiglaucoma therapy. It is well established that a water-soluble sulfonamide, also possessing relatively balanced lipid solubility, would be an effective antiglaucoma drug via the topical route (Supuran, C.T., et al. *Expert Opin. Ther. Patents* **2002**, *12*, 217–242; Supuran, C.T., et al. *Med. Res. Rev.* **2003**, *23*, 146–189).

Design of new aromatic sulfonamides was carried out using computational methods of theoretical medicinal chemistry as described in our previous works (Remko, M., et al. *Bioorg. Med. Chem.* **2004**, *12*, 5395–5403). Of particular interest are the molecular geometries of neutral and anionic species, acidities, and lipophilicities. Synthesis of designed new aromatic sulfonamides was conducted according to the procedures described in the references. Antiglaucoma activity of the designed compounds was evaluated in both in vitro and in vivo conditions. For determination of the intraocular pressure changes experiments with adult male chinchilla rodents were performed. In this contribution we present the design and synthesis of a novel drug-like aromatic sulfonamides of general formulas I and II with favorable biological, structural, physicochemical and some pharmacokinetic properties comparable to those obtained for the therapeutically useful acetazolamide, dorzolamide and brinzolamide. The solid-state structure of novel aromatic sulfonamides has been examined by X-ray crystallography. Theoretical medicinal chemistry methods were applied for structural characterization of these compounds in the gas phase and water solution. Of particular interest are the molecular geometries of neutral and anionic species, acidities, and lipophilicities. The data obtained allows us to assume that new aromatic sulfonamides may represent a novel class of compounds in the search for new effective antiglaucoma drugs (Supuran, C.T., et al. *Int. J. Enzyme Inhib. Med. Chem.* **2013**, *28*, 289–293*;* Gregáň, F., et al. *US patent No. 8193184*, 5 June 2012).


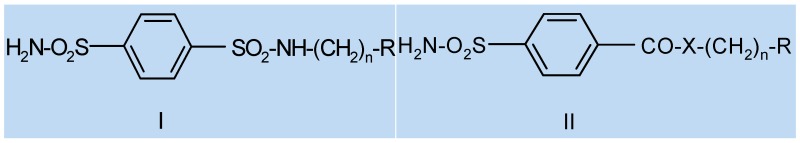


### 2.9. Design and Synthesis of Macrocyclic Scaffolds for Compounds with Potential Antituberculosis/Antibacterial Activity and Improved CYP450 Properties (A013)

VasilevichNatalya I.*Aksenova (Naimark)Elena A.AksenovaAnna A.AfanasyevIlya I.“Novie Nauchnie Technologii” Ltd. (ASINEX group), 20 Geroev Panfilovtsev, Moscow 125480, Russia*Correspondence: nvasilevich@asinex.com

Based on an earlier suggested general pharmacophore approach, compounds active against both tuberculosis virulent strain *H37Rv* and *Staphylococcus aureus* were obtained. The main drawback of these compounds was an almost complete CYP 3A4 inhibition, which presumably could be overcome by macrocyclization. Two 15–16-membered macrocyclic scaffolds C and D shown in the picture with pharmacophore groups located in appropriate positions were designed and synthetic schemes for their achievement were elaborated.


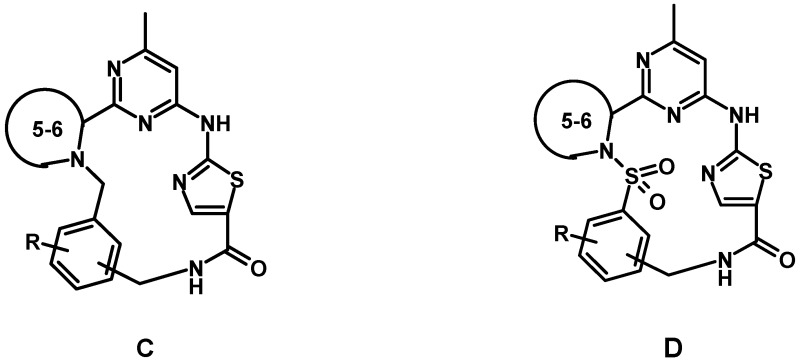


Testing of these compounds against CYP450 enzymes confirmed generally lower inhibition of key cytochromes CYP 3A4 and CYP2D6 by suggested macrocyclic compounds compared to acyclic prototypes. New macrocyclic compounds retained the ability to inhibit *Staphylococcus aureus* growth. The best compound XI showed low CYP450 inhibition and significant (77.2%) inhibition of *Staphylococcus aureus* growth.

**Acknowledgments:** This work was supported by the Ministry of Education and Science of the Russian Federation (agreement 14.576.21.0019 dated 27 July 2014).

### 2.10. Studying the Influence of Stereochemistry in P-gp Modulation: Case-Study with Thioxantones (A014)

LopesAna^1^MartinsEva^2^SilvaRenata^2^FernandesCarla^1^,^3^PalmeiraAndreia^1^RemiãoFernando^2^PintoMadalena M. M.^1^,^3^SousaEmília^1^,^3^*1Laboratório de Química Orgânica e Farmacêutica, Departamento de Ciências Químicas, Faculdade de Farmácia, Universidade do Porto, Rua Jorge Viterbo Ferreira 228, 4050-313 Porto, Portugal2REQUIMTE, Laboratório de Toxicologia, Departamento de Ciências Biológicas, Faculdade de Farmácia, Universidade do Porto, Rua Jorge Viterbo Ferreira 228, 4050-313 Porto, Portugal3Centro Interdisciplinar de Investigação Marinha e Ambiental (CIIMAR/CIMAR), Universidade do Porto, Rua dos Bragas 289, 4050-123 Porto, Portugal*Correspondence: esousa@ff.up.pt

Chirality is an interesting geometric property and it is meaningful to explore the interactions between chiral small molecules and stereoselective biomacromolecules with pre-clinical and clinical significance. Since the first observation of enantioselective binding to human-derived P-glycoprotein (P-gp) by mefloquine enantiomers (Lu, L., et al. *Pharm. Res.* **2001**, *18*, 1327–1330), sparse stereoselectivity studies with P-gp modulators have emerged. Recently, we have shown that newly synthesized (thio)xanthonic derivatives protect against toxic P-gp substrates acting as potent inducers/activators of this transporter (Silva, R., et al. *Arch. Toxicol.* **2015**, *89*, 1783–1800). Now we aim to discover new P-gp modulators and enlightening the stereoselectivity of this ABC transporter face to this class of modulators. Herein, we report the synthesis and characterization of a library of new chiral aminated thioxanthones in their enantiomeric pure form and in silico and in vitro preliminary results concerning their P-gp modulation behavior.


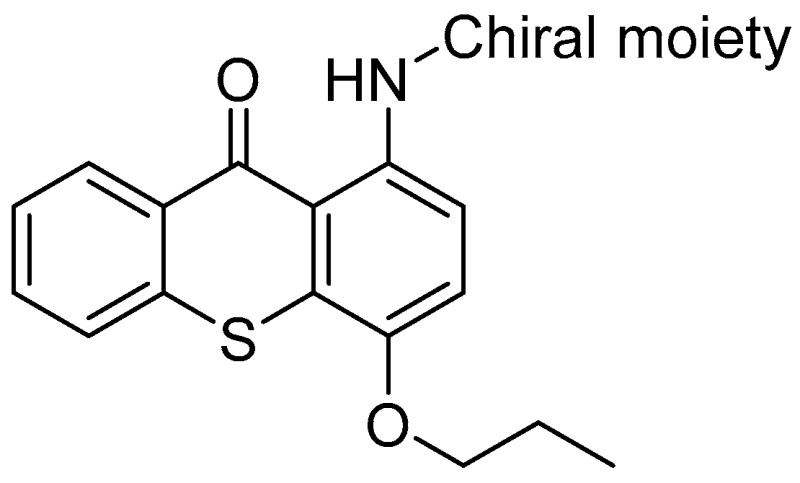


In silico docking studies in P-gp rat model anticipated enantioselectivity for these new derivatives. The thioxanthones’ cytotoxicity was evaluated by the Neutral Red uptake assay, in order to select a non-cytotoxic working concentration. The compounds were assessed for their influence in P-gp ATPase assay. The investigation of P-gp expression and activity allowed to discover new P-gp modulators. Nevertheless, no significant differences between enantiomeric pairs of thioxanthones were observed.

**Acknowledgments:** This work was partially supported through national funds provided by: FCT—Foundation for Science and Technology and European Regional, Development Fund (ERDF) and COMPETE under the projects PEst-C/MAR/LA0015/2013, PTDC/MAR-BIO/4694/2014, and INNOVMAR—Innovation and Sustainability in the Management and Exploitation of Marine Resources, reference NORTE-01-0145-FEDER-000035, Research Line NOVELMAR.

### 2.11. Plasma Neurotransmitters Variation in Growth Hormone Deficient Children Under rh-GH Replacement Therapy. Preliminary Data (A015)

StefanescuAna-Maria*DumitrescuCristinaPadureAdrianaNational Institute of Endocrinology “C. I. Parhon”, Bucharest, Romania*Correspondence: stefanescuam@yahoo.com

*Aim*: To evaluate the impact of rh-GH replacement therapy on neurotransmitters: gamma—amino butyric acid (GABA), dopamine (DA) and serotonin (5-HT) in growth hormone deficient children (Schneider, H.J*.*, et al. *Eur. J. Endocrinol.* **2003**, *149*, 377–392*;* Ayuk, J., et al. *Postgrad. Med. J.* **2006**, *82*, 24–30).


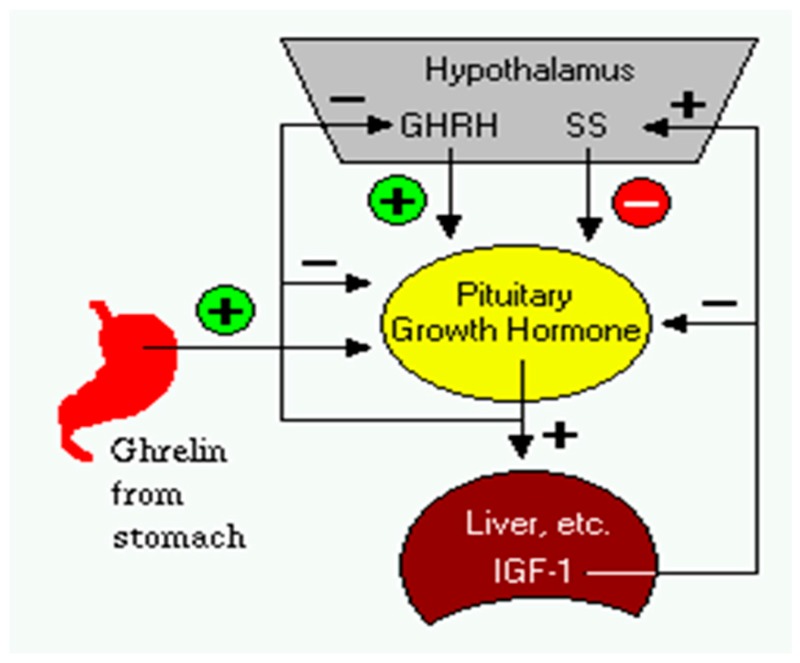


*Research design and methods:* This retrospective study included 30 subjects with growth hormone deficit clinically established: 20 boys (5–14 years) and 10 girls (6–14 years). All of them underwent GH replacement therapy from 9 months–10.6 years. rh—GH dose varied in all subjects from 0.6–1.9 mg/day based on detailed clinical and anthropometric data. In 2015, all subjects in different phases of treatment were tested for plasma: GABA, DA, 5-HT and IGF-1.

*Results*: Median plasma GABA in boys vs. girls was: 50.5 vs. 46 ng/mL; median plasma DA in boys vs. girls was: 43.34 vs. 29.4 pg/mL; median 5-HT in boys vs. girls was: 227.5 vs. 208.7 ng/mL and median IGF-1 in boys vs. girls was: 334 vs. 357.25.7 ng/mL. We established a statistically significant difference in plasma GABA and in DA values in boys vs. girls. High multiple regression coefficients were established between age and IGF-1, DA, GABA or between IGF1 and 5-HT, DA, GABA in boys vs. girls.

*Conclusions*: This study established a link between brain neurotransmitters and the height gain in GH-deficient children under replacement therapy in different phases of treatment.

### 2.12. Tyrosine Hydroxylase, the Rate-limiting Enzyme in Catecholamine Biosynthesis Could Be an Index of Functionality in Pheochromocytoma Diagnosis (A016)

StefanescuAna-Maria^1^*SchiporSorina^1^BadiuCorin^1^,^2^1National Institute of Endocrinology “C. I. Parhon”, Bd. Aviatorilor, 34–36, Bucharest, Romania;2University of Medicine and Pharmacy “Carol Davila”, Bd. Eroilor Sanitari, 8, Bucharest, Romania*Correspondence: stefanescuam@yahoo.com

*Aim*: Excess release of catecholamines is a characteristic of pheochromocytomas. The rate of catecholamine synthesis is determined by tyrosine hydroxylase (TH) enzyme. (Nagatsu, T., et al, *J. Neural. Transm.* (Vienna), **2016**, *123*, 1255–1278). Usually, TH is detected by immuno-histochemistry. In our study, we evaluated this enzyme in plasma from pheochromocytoma subjects establishing correlations with some metabolites.


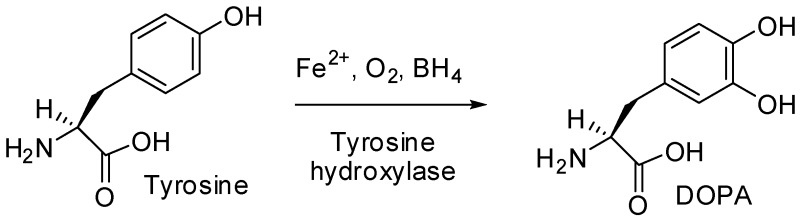


*Subjects and Methods*: Ten subjects (nine women/one man aged 40–72 years) clinically suspected of pheochromocytoma, were biochemically investigated for free plasma normetanephrines (NMNp)/metanephrines (MNp), plasma chromogranin A (CgA), plasmaTH. Comparison of tumoral metabolites values were done with our lab normal range Statistical analysis used multiple regression to evaluate relationship between TH and all three parameters: NMNp/MNp/CgA. 

*Results*: All subjects showed excessive plasma NMNp (median: 1434 pg/mL); in seven cases there was an oversecretion of MNp (median: 441 pg/mL) and CgA was higher in nine cases (median: 668 ng/mL).TH was identified in all plasma samples with a median of: 2.08 ng/mL. Greater TH values were detected in cases with an overexcess of metabolites. Good correlations were established between NMNp/TH (0.51), MNp/TH(0.81)NMNp/CgA (0.71).

*Conclusions*: Metabolites detected in excess correlate well with TH values, proving a great rate of catecholamine synthesis in some cases. We could affirm TH could be used as an index of functionality in pheochromocytoma diagnosis.

### 2.13. TRP Modulators Based on Glycine and Mono-, Bicyclic Terpenoids—Synthesis and Pharmacological Properties (A017)

NesterkinaMariia*KravchenkoIrynaDepartment of Pharmaceutical Chemistry, Odessa National University, Odessa 65026, Ukraine*Correspondence: mashaneutron@gmail.com

Currently, significant interest in drug development is focused on obtaining drugs that simultaneously affect various pharmacological targets and thus exhibit combined actions. Herein we demonstrate the possibility of development of novel drugs that are able to simultaneously modulate TRP-channels and bind to glycine receptors. For this purpose esters based on mono- and bicyclic terpenoids (menthol, thymol, carvacrol, guaiacol, eugenol, borneol) and an inhibitory amino acid (glycine) were synthesized via Steglich esterification. Their anticonvulsant action was evaluated by a PTZ-induced convulsion model and analgesic effect—by pharmacological models of thermal and chemical stimuli. All studied esters were found to produce antinociceptive effects and attenuate acute pain more than the reference drug benzocaine after topical application. The present findings indicate that glycine esters of the abovementioned terpenoids are not classical prodrugs and possess their own pharmacological activity. Prolonged antiseizure action of the esters was revealed at 24 h after oral administration. Moreover, orally co-administered gidazepam (1 mg/kg) and glycine esters produce synergistic seizure prevention effects.


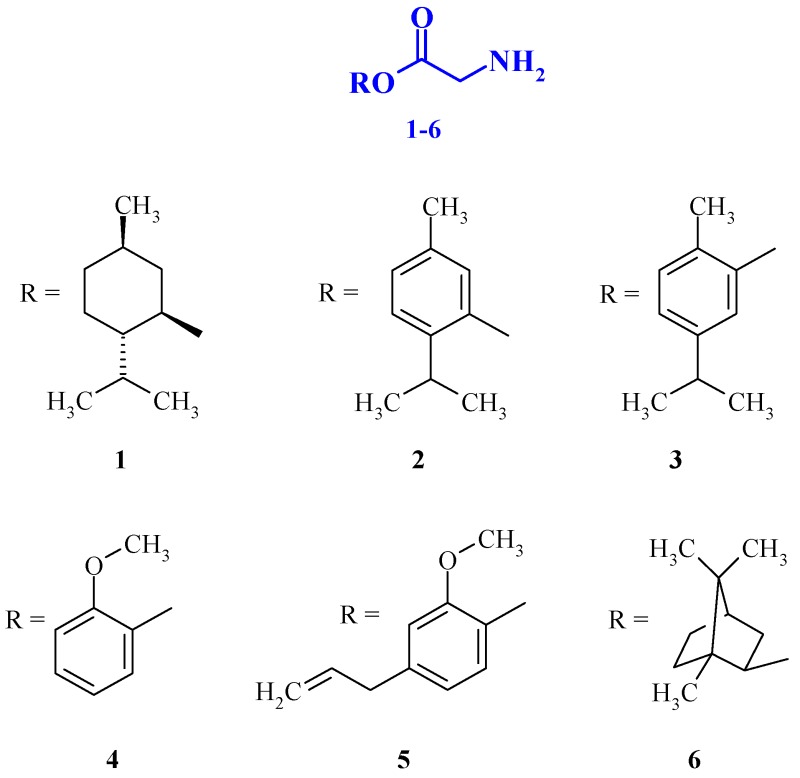


### 2.14. Antiproliferative Activity and Effect on GABA_A_ Receptors of the Abietane Diterpenoid Jiadifenoic Acid C and Other Callitrisic Acid (4-Epidehydroabietic Acid) Derivatives (A018)

González-CardeneteMiguel A.^1^*StadlerMarco^2^PadrónJosé M.^3^1Instituto de Tecnología Química (UPV-CSIC), Universitat Politècnica de Valencia-Consejo Superior de Investigaciones Científicas, Avda de los Naranjos s/n, 46022 Valencia, Spain2Department of Pharmacology and Toxicology, University of Vienna, Althanstrasse 14, A-1090 Vienna, Austria3BioLab, Instituto Universitario de Bio-Orgánica “Antonio González”, Centro de Investigaciones Biomédicas de Canarias, Universidad de La Laguna, C/Astrofísico Francisco Sanchez 2, La Laguna 38200, Tenerife, Spain*Correspondence: migoncar@itq.upv.es

The abietane-type and related diterpenoids are a class of naturally occurring terpenoids found in the plant kingdom, which have demonstrated a wide range of biological activities against cancer, and a variety of infectious diseases (both viral and bacterial) (González, M.A., *Nat. Prod. Rep.* **2015**, *32*, 684–704). For example, dehydroabietic acid (DHA, **1**), displays not only antiulcer and antimicrobial properties, but also antitumor effects. Recently, DHA was reported as a positive GABAA receptor modulator (potentiation of I_GABA_ 682.3% at 100 μM) (Rueda, D.C. et al. *Fitoterapia* **2014**, *99*, 28–34).


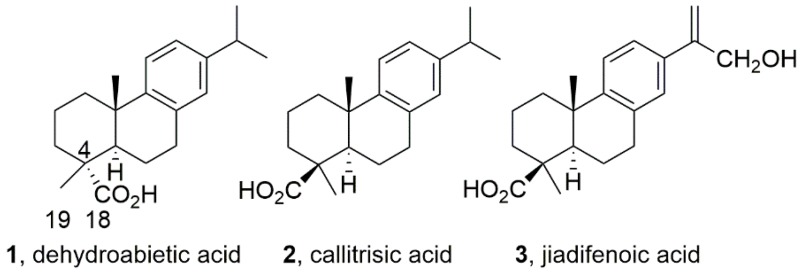


DHA displays an equatorial carboxylic group located at C18 while in other natural congeners the carboxylic group adopts the axial configuration (C19) as in 4-epidehydroabietic acid or callitrisic acid (**2**). Recently, a series of related acids having a C19 carboxylic group have been isolated. For example, the jiadifenoic acids A-I. These have shown antiviral properties against Coxsackie virus. We have developed the synthesis of jiadifenoic acid C (**3**) from callitrisic acid isolated from Sandarac resin (González, M. A. et al. *J. Nat. Prod.* **2014**, *77*, 2114–2117). The ready availability of these acids from our work and the absence of their biological and pharmacological studies as well as chemical manipulation prompted us to carry out this research. In this communication, we report on the synthesis of several derivatives of callitrisic acid along with the results of the evaluation of their antiproliferative and modulating GABAA receptor activities.

**Acknowledgments:** Financial support by the Spanish Government [Consejo Superior de Investigaciones Científicas (201680I008)] is gratefully acknowledged. M.S. thanks the support by the doctoral program “Molecular Drug Targets” (Austrian Science Fund FWF W 1232).

### 2.15. Antiviral Activity of Fluorinated Heterocyclic Compounds (A019)

BiliavskaLiubov^1^*PankivskaYulia^1^PovnitsaOlga^1^ZagorodnyaSvitlana^1^GudzGanna^2^PikunNadiia^2^ShermolovichYuriy^2^1Institute of Microbiology and Virology, National Academy of Sciences of Ukraine, Zabolotnogo str., 154, Kyiv 03680, Ukraine2Institute of Organic Chemistry NAS of Ukraine, Murmanska Str., 5, Kyiv 02660, Ukraine*Correspondence: bilyavskal@ukr.net

Human adenoviruses (HAdV) are ubiquitous infectious DNA viruses possessing a broad spectrum of pathogenicity. More than 60 HAdV serotypes have been identified that are responsible for different respiratory, gastrointestinal, and ocular diseases. HAdV are able to persist in humans for a long time in a latent state and can be reactivated by various factors. They cause grave problems in immunocompromised hosts by the development of generalized HAdV infection. The diseases caused by Herpes Simplex virus are widely distributed. Treatment of these infections is the most significant medical problem. However, the appearance of resistant virus is a current problem in the treatment of patients and deficiency in the antiviral preparations caused their toxicity. Therefore, it is very important to develop new antiviral drugs against this virus. 

Nucleoside analogues have a special place among the most effective antiviral drugs. The study of fluorinated nucleoside sugars chemistry became the background for the development of promising chemotherapeutic agents with antitumor and antiviral effects. Compounds with modified heterocyclic core compared with the natural nucleoside analogues are now of interest. Our research has been related to the determination of cytotoxicity and antiviral activity regarding HSV-1, HSV-2 and HAdV5 of new fluorinated compounds possessing different chemical structures.


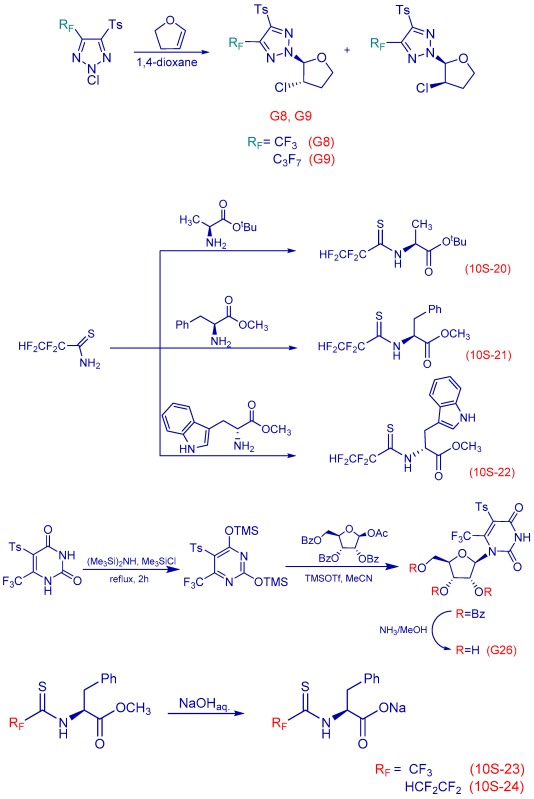


Cytotoxicity of the compounds was determined by MTT-test or neutral red dye (NR) according to standard protocols. Antiviral effect was determined using PCR, MTT-test and cytomorphological methods. 

Two compounds, G8 and 10S-23, inhibited the reproduction of all viruses with different efficiency. Compound G9 was active only in relation to both types of herpes. G26 suppressed reproduction of HSV-2/BH and HAdV5, whereas compounds 10S-20 and 10S-24 were active only in relation to reproduction HSV-1/US. According to the results obtained in the present study: antiviral activity of substances submitted to the post-treatments was demonstrated; the absence of antiviral activity of substances submitted to the pre-treatments, during absorption and penetration of the virus was shown; absence of virucidal activity of substances was shown. That data suggest that the substances may be active on the latter stages of virus reproduction. The results are evidence of promising compounds for further in-depth study on models in vitro and in vivo.

### 2.16. Design and Development of 9,10-Dihydrophenanthrene Derivatives as Inhibitors of Human Protein Kinase CK2 (A020)

HaidarSamer*MeyersAnnikaJoseJoachimInstitut für Pharmazeutische und Medizinische Chemie, PharmaCampus, Westfälische Wilhelms-Universität Münster, Corrensstr. 48, 48149 Münster, Germany*Correspondence: shaid_01@uni-muenster.de

Casein Kinase 2 (CK2) is a ubiquitous heterotetrameric eukaryotic serine/threonine protein kinase. CK2 has important roles in different cellular functions. This enzyme enhances cancer phenotype by blocking apoptosis and stimulating cell growth. Thus, inhibition of CK2 can induce the physiological process of apoptosis leading to tumor cell death. Large number of compounds has been developed as kinase inhibitors including CK2 inhibitors. Most of the CK2 inhibitors contain a heterocyclic backbone which fits into the active site of the CK2α subunit and competes with the ATP. Recently we reported on the development of compound 3: di 2,6-Di(furan-3-yl)anthracen-9,10-dion as an active CK2 inhibitor (Haidar, S., et al. *Pharmazie* **2015**, *70*, 772–776).


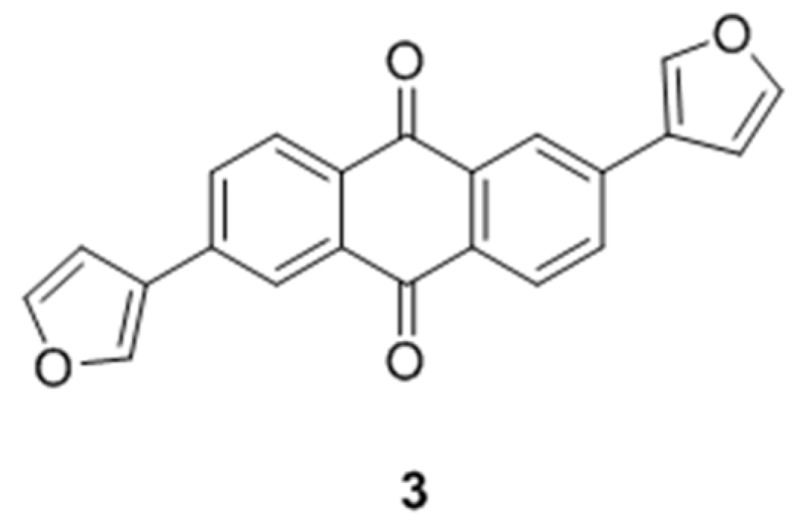


With the aim of finding new inhibitors for this target a series of di-substituted 9,10-dihydrophenanthrene was designed as CK2 inhibitors and docked in the crystal structure of the enzyme to evaluate their ability to fit in the active site.


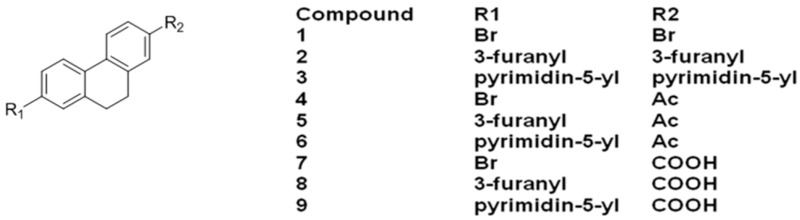


The main idea was to explore if the 9,10-dihydrophenanthrene backbone is more suitable for binding than the backbone of compound **3** and to determine if the introduced functional groups can form hydrogen bonds with the amino acid residue. Some of the designed compounds were synthesized and tested in vitro as well. The docking study clearly indicates that the designed compounds fit well in the ATP active site of the enzyme, and most of them were able to create hydrogen bonds with some amino acids residues.

### 2.17. Indeno[1,2-b]indole Inhibitors of Human Protein Kinase CK2 and Their Impact on Different Tumor Cell Lines (A021)

AicheleDagmar^1^*Le BorgneMarc^2^JoseJoachim^1^1Institute of Pharmaceutical and Medicinal Chemistry, Westfälische Wilhelms-Universität, PharmaCampus, Corrensstraße 48, 48149 Münster, Germany2ISPB-Faculté de Pharmacie, Université Claude Bernard Lyon 1, EA 4446 Bioactive Molecules and Medicinal Chemistry, 8 avenue Rockefeller, 69373 Lyon cedex 08, France*Correspondence: dagmar.aichele@uni-muenster.de

Increased protein kinase CK2 activity is involved in many human diseases such as cancer (Guisiano, S., et al. *Eur. J. Cancer* **2011**, *47*, 792–801). In consequence CK2 is an emerging major target for drug design. Several indeno[*1*,*2*-*b*]-indole-9,10-dione derivatives containing N^5^-isopropyl substitutions on the C-ring were synthesized and have been reported as potent ATP-competitive CK2 inhibitors (Alchab, F., et al. *Pharmaceuticals* **2015**, *8*, 279–302; Gozzi, J.G., et al. *J. Med. Chem.* **2015**, *58*, 265–277).

Here we report on the evaluation of these inhibitors, containing different substituents in the A- and D-rings, for their effects on various tumor cell lines: breast cancer cells MCF-7, lung carcinoma cells A427 and epidermal cancer cell line A431. The most potent CK2 inhibitor contains an O-prenyl residue R_1_ and exhibits an IC_50_ value of 0.025 µM. Treatment of MCF-7 cells with 20 µM of that compound for 24 h results in a reduction of the total cell number by 90%. Most of the remaining cells exhibited apoptotic morphology and showed nearly none proliferating activity. In contrast treatment of A431 cells and A427 cells caused only a moderate decrease of cell proliferation by 30% for all tested compounds.

This study shows that potent CK2 inhibitors as tested can exhibit distinct effects on different tumor cell lines. Compound containing an O-prenyl residue R_1_ appears to be an antiproliferation agent with high activity toward MCF7 cells.


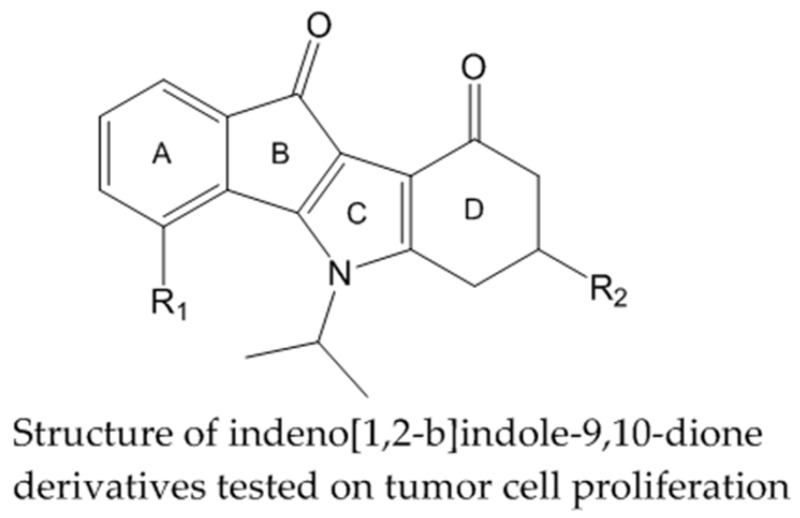


### 2.18. TLR4-Mediated Activation of Monocytes by Human αS1-Casein (A022)

SaengerThorsten^1^*VordenbäumenStefan^2^TahanTamara^1^NienbergChristian^1^BleckEllen^2^SchneiderMatthias^2^JoseJoachim^1^1Institute of Pharmaceutical and Medicinal Chemistry, Westfälische Wilhelms- Universität, PharmaCampus, Corrensstraße 48, 48149 Münster, Germany2Policlinic of Rheumatology, Hiller Research Unit, University Clinic Duesseldorf, Heinrich-Heine-University Düsseldorf, Düsseldorf, Germany*Correspondence: Thorsten.saenger@wwu.de

Human milk protein αS1-casein (CSN1S1) was shown to be overexpressed in autoimmune diseases (OA, BPH, MS) as well as in cancer. CSN1S1 displays opioid-like activity and modulates the innate immune response of intestinal cells. Recently, it was demonstrated that CSN1S1 induces the expression of proinflammatory cytokines (IL 1β and IL 6) in monocytic cells via MAPK-p38 signaling (Vordenbäumen, S., et al. *BMC Immunol*. **2013**, *14*, 46; Vordenbämen, S., et al. *J. Immunol*. **2011**, *186*, 592–601).

In this study the human TLR4 receptor, a receptor of the innate immune system, was identified as an interaction partner of human CSN1S1 inducing expression of cytokines IL 1β, IL 6 and IL 8 in human monocytic cells concentration- and time-dependently (Vordenbäumen, S., et al. *Mol. Nutr. Food Res*. **2016**, *60*, 1079–1089). In HEK293 cells cotransfected with TLR4 human CSN1S1 (purified from Escherichia coli) induced secretion of chemokine IL-8 secretion. In vitro flow cytometric assay confirmed CSN1S1—TLR4 receptor interaction. Chemokine sectretion as well as binding was not detected for CSN1S1 phosphorylated by protein kinase CK2 as well as denaturated CSN1S1. This supports the hypothesis, that CSN1S1 is a ligand of the TLR4 receptor exerting proinflammatory properties in phosphorylation-dependent manner. In conclusion CSN1S1 could contribute to the development of a potent immune system in breastfed offspring.


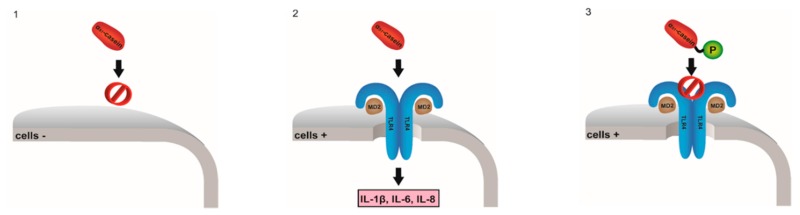


**Acknowledgments:** The authors gratefully acknowledge financial support of this study by a grant from the Hiller Rheumatology Research Foundation, Erkrath, Germany and Hiller Research Center Rheumatology of Heinrich-Heine-University Düsseldorf, Germany.

### 2.19. Lead Selection of Antiparasitic Compounds from a Focused Library of Benzenesulfonyl Derivatives of Heterocycles (A024)

PaglieroRomina J.^1^KaiserMarcel^2^,^3^BrunReto^2^,^3^NietoMarcelo J.^4^*MazzieriMaría R.^1^1Departamento de Farmacia, Facultad de Ciencias Químicas, Universidad Nacional de Córdoba. Córdoba, Argentina (Present address: Department of Cell Biology, Cell Screening Centre, University Medical Centre Utrecht, 3584 CX Utrecht, The Netherlands)2Department Medical Parasitology and Infection Biology, Swiss Tropical Institute, CH-4002 Basel, Switzerland3University of Basel, CH-4003 Basel, Switzerland4Department of Pharmaceutical Sciences, School of Pharmacy, Southern Illinois University Edwardsville, Campus Box 2000, Edwardsville, IL 62026, USA*Correspondence: mnieto@siue.edu

A library of 69 synthetic benzenesulfonyl derivatives of heterocycles, with drug-like properties, was assayed for in vitro antiparasitic activity and the results were added to our previous report for a comprehensive SAR discussion. Seven compounds showed an IC_50_ between 0.25–3 μM against *L. donovani* and low cytotoxicity.

Compound 1-(2,3,5,6-tetramethylphenylsulfonyl)-2-methylindoline (**G16**), was particularly interesting, with an IC_50_ similar to the reference drug miltefosine. In addition, seven compounds showed an IC_50_ below 6 µM against *T. cruzi*, and three of them (**E3**, **E9** and **G3**) were identified as lead scaffolds for further optimization based on their activity-toxicity profile. Furthermore, two promising structures (**B15** and **G15**) have shown moderate inhibitory activity against *P. falciparum*. In general, the presence of a benzenesulfonyl moiety improves the antiparasitic activity of the heterocycles included in this study (with exception of *T. b. rhodesiense*) validating the criteria used in the selection of the fragment-based drug design approach. Finally, from the SAR analysis it could be concluded that the presence of electron withdrawing and lipophilic groups were favorable for the antiparasitic activity.


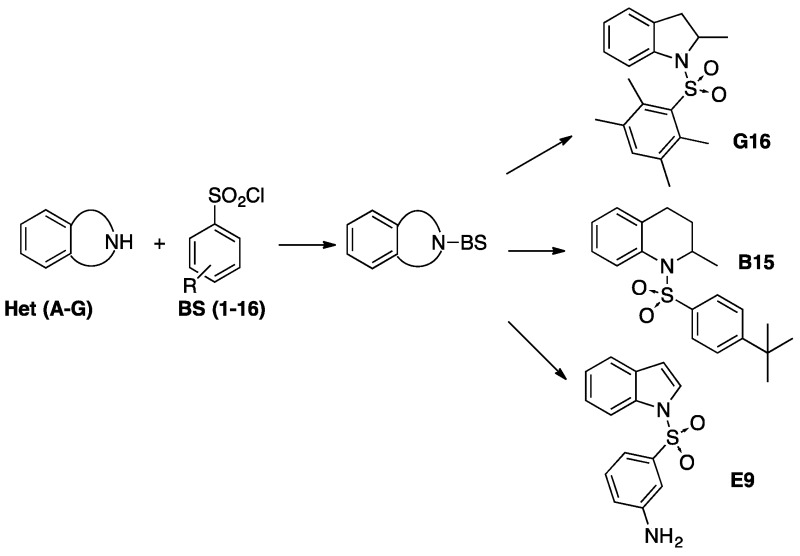


**Acknowledgments:** This work was financially supported by Southern Illinois University Edwardsville, Multidisciplinary Research Team award 2008-2010 and by SECyT-UNC (Argentina). R.J.P. would like to thank to CONICET fellowship and the Council for International Exchange of Scholars and Bunge-Born Foundation for her Fulbright-Bunge Born Scholarship fellowship.

### 2.20. Antibacterial and Antibiofilm Screening of New Platinum (IV) Complexes with some S-Alkyl Derivatives of Thiosalicylic Acid (A027)

MijajlovićMarina^1^VasićSava^2^RadojevićIvana^2^*MaksimovićJovana^2^ČomićLjiljana^2^NikolićMiloš^1^RadićGordana^1^1Faculty of Medical Sciences, University of Kragujevac, S. Markovića 69, 34000 Kragujevac, Serbia2Department of Biology and Ecology, Faculty of Science, University of Kragujevac, R. Domanovića 12, 34000 Kragujevac, Serbia*Correspondence: ivana@kg.ac.rs

This investigation showed influence of five new Pt(IV) complexes on 16 strains of bacteria. Antibacterial activity was tested using microdilution method with resazurin while antibiofilm activity was observed by tissue culture plate method and antibiotic doxycycline was used as positive control. The results were expressed as minimum inhibitory concentration (MIC), minimum bactericidal concentration (MBC) and biofilm inhibitory concentration (BIC). The complexes were labeled from **C1** to **C5**. The best result on Gram positive bacteria was obtained with **C1** and MIC on *Staphylococcus aureus* ATCC 25923 was ˂7.81 μg/mL. *Bifidobacterium animalis subsp. lactis* (probiotic) was sensitive to **C2** (MIC at 15.625 μg/mL). The best sensitivity on Gram negative bacteria was observed on *Escherichia coli* ATCC 25922 with **C1**, **C2**, **C3** and **C4**, on *Proteus mirabilis* ATCC 12453 with **C1**, and on *Pseudomonas aeruginosa* with **C2**, **C3** and **C5** (all MICs at 250 μg/mL). The tested complexes were more efficient as antibiofilm agents and the best results were obtained with **C2** acting against *S. aureus* and *S. aureus* ATCC 25923 biofilm. In conclusion, we noticed that the tested compounds exhibited promising properties as antibacterial agents and antibiofilm agents.

**Acknowledgments:** This work was supported by the Ministry of Education, Science and Technological Development of the Republic of Serbia.

### 2.21. Investigation of Acute Toxicity of Tilorone Ointment for Topical Treatment of Herpes Virus Infection (A028)

VashchenkoOksana^1^*BabiyOlena^2^ZholobakNadiya^3^VashchenkoKateryna^1^1Danylo Halytsky Lviv National Medical University, Lviv, Ukraine2Vinnytsya Medical College named after Academician D.K. Zabolotny, Vinnytsya, Ukraine3D.K. Zabolotny Institute of Microbiology and Virology, Kyiv, Ukraine*Correspondence: o_vashchenko@ukr.net

Tilorone (amixin, dihydrochloride 2,7-bis[2-(diethylamino)ethoxy] fluorenone-9) is a low molecular interferon inducer that is effective against a wide range of viral infections including herpes viruses. The mechanism of tilorone antiviral action is associated with inhibition of virus-specific peptides translation in infected cells resulting in the suppression of virus replication. An appropriate therapy of the viral infections with external rash besides the systemic preparations must include topical drugs that will reduce clinical signs of infection, improve skin epithelialization, and decrease time of virus elimination. Considering this, we developed a new topical antiviral 2% tilorone preparation in the form of ointment.

The aim of investigation was to study the parameters of acute toxicity of the 2% tilorone ointment. In accordance with the requirements of the European Pharmacopoeia 8.0 the acute toxicity of the developed ointment was studied using two methods—intragastric administration and cutaneous application. All animals were treated in compliance with the European Convention for the Protection of Vertebrate Animals used for Experimental and Other Scientific Purposes [Council of Europe, Strasbourg, 2006].

Acute intragastric toxicity was carried out in two species of rodents [54 white outbred mice (20–25 g) and 27 white Vistar rats (200–220 g)] that were given intragastrically an aqueous solution of ointment at a dose of 0.5 g/kg. Acute dermal toxicity was studied in 27 white outbred mice (20–25 g); the application area was 5.5–6.0 cm^2^.

The animals were monitored daily for clinical signs (breathing, physical activity, convulsions, analgesia, muscle tone, ophthalmic, cardiovascular and gastrointestinal symptoms, diuresis, skin signs) in the course of 14 days. All the animals remained alive and active; the distinctive symptoms of intoxication were absent. It was also determined that an effective dose of 2% test ointment does not cause toxic effects on human body is DL_0_ ≤ 0.4 g/kg.

### 2.22. Antiviral and Apoptosis Moduling Potential of Fluorinated Compounds (A029)

NaumenkoKrystyna^1^*GolovanAnna^1^BaranovaGalina^1^ZagorodnyaSvitlana^1^ShermolovychYurii^2^1D.K. Zabolotny Institute of Microbiology and Virology NAS of Ukraine, 154 Acad. Zabolotny str., Kyiv, Ukraine2Institute of Organic Chemistry NAS of Ukraine, 5, Murmanska Str., Kyiv, Ukraine*Correspondence: krystyn.naumenko@gmail.com

In our laboratory, we focus on the design, synthesis, and evaluation of fluorinated compounds that could be used in the treatment of diseases caused by the Epstein-Barr virus (EBV) and virus vesicular stomatitis (VVS) (Peng L., et al. *J. Fluor. Chem.* **2008**, *29*, 743–766). We used the model of DNA and RNA viruses, which are characterized by acute and latent clinical form of the disease.

For this purpose, in a first step, we used the PASS software by which we could identify potential antiviral candidates among which a fluorinated derivative of uracil (G27), a trifluoromethyl-substituted derivative of a thiosugar (SBIO6), a bisphosphonic acid (10s19) and several derivatives of alanin and glucose (10s20–10s28) (Lagunin A., et al. *Bioinformatics* **2000**, *16*, 747–748).

**Table:** 

G27	10s20	10s19
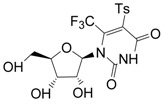	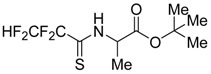	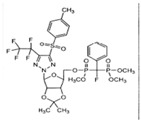
SBIO6	10s24	10s25
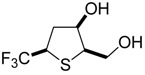	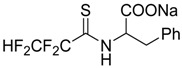	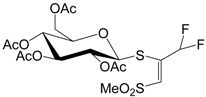

Our in vitro studies revealed an antiviral activity for a few compounds. A real time polymerase chain reaction (PCR) assay was performed to assess the antiviral activity of various drugs against EBV. The MTT-assay was used for detection anti-VVS activity. Three compounds appeared to be effective against EBV: **G27** (EC_50_ = 100 µg/mL), **10s20** (EC_50_ = 1 µg/mL), and **10s25** (EC_50_ < 62 µg/mL, replication of EBV was suppressed at 100% at a concentration of 62 µg/mL). The ability to inhibit reproduction of VVS was shown for compound **10s19** (EC_50_ = 19 µg/mL) and for compound **10s24** (EC_50_ = 29 µg/mL). It was established that the selectivity indices of these compounds ranged from 10 to 100.

As Epstein-Barr virus (EBV) is the cause of several lymphoproliferative diseases, we studied the apoptosis induction potency of compounds **G27** and **SBIO6** (Novalic Zlata, et al., *Med. Chem*. **2016**, *6*, 449–466). Apoptotic cells were detected using a flow cytometry (Riccardi C., et al. *Nat. Protoc*. **2006**, *1*, 1458–1461). The sub-G1 peak was measured with a Epics XL flow cytometer (Beckman Coulter, Brea, CA, USA) and analyzed using Flowing Software, version 2.5. Addition of **G27** led to the observation of two peaks in the histogram, what may indicate a break of the cell cycle by this compound. It was also established that by addition of **SBIO6** the percentage of apoptotic cells was significantly increased when compared to the control and reached 70%–90%. The obtained data let us regard these compounds as perspective antiviral and antitumor agents. Combination of predictive analysis with in vitro studies allows to evaluate the biological properties of compounds and to discover the mechanism of their action.

### 2.23. Heat Shock Proteins in Target Specific Cancer Chemotherapy (A030)

TutarYusuf^1^*ÖzgürAykut^2^1Division of Biochemistry, Department of Basic Sciences, Faculty of Pharmacy, Cumhuriyet University, Sivas, Turkey2Department of Bioengineering, Faculty of Natural Sciences and Engineering, Gaziosmanpaşa University, Tokat, Turkey*Correspondence: ytutar@cumhuriyet.edu.tr

Heat shock proteins (Hsps) are important biological targets in next generation cancer treatment. Hsps play vital roles in protein hemostasis pathways (proper folding and stabilization of nascent proteins, inhibition of protein aggregation, degradation of aggregated proteins, signal transduction and protein translocation) in eukaryotic and prokaryotic cells. Hsps are found in different cellular compartments and their expression level is increased in response to cellular and external stress factors (tumorogenesis, UV light, hypoxia, oxidative, infection, stress, fever, temperature variation) (Tutar, L, et al., *Curr. Pharm. Biotechnol.* **2010**, *11*, 216–222). Therefore, pathogenesis of diseases is related with expression level of the Hsps. Hsps are over-expressed in cancer cells, and especially, Hsp27, Hsp70 and Hsp90 are involved in all phases of tumorogenesis (apoptosis, metastases, angiogenesis, invasion, and cell differentiation). Hsp27, Hsp70 and Hsp90 ensure stabilization, activation and proper folding of the oncogenic proteins in cancer cells. Therefore, inhibition of Hsps has been significant therapeutic strategy for next generation target specific cancer treatment. Inhibition of Hsp90 chaperone activity has been significant drug target for the past 30 years in cancer treatment. Now, approximately 20 different compounds are in clinical phase studies (Ozgur, A, et al., *Curr. Proteol.* **2014**, *11*, 2–16; Ozgur, A., et al., *Anticancer Agents Med. Chem.* **2016**, *16*, 280–290). Clinical and pre-clinical studies demonstrated that, inhibition of Hsp90 activity is not enough by itself. Inhibition of Hsp90 triggers expression of Hsp70 and complements inhibited Hsp90 chaperone activity. Moreover, Hsp27 controls and regulates key points of the apoptotic pathway in cancer cells. Therefore, in addition to Hsp90 inhibition, blocking of Hsp70 and Hsp27 chaperone activities have been remarkable therapeutic strategy for cancer treatment (Tutar, Y. *Adv. Tech. Biol. Med.* **2015**, *3*, 1000e109).

In our lab, we designed and synthesized novel pyrimidine and coumarine derivative compounds as Hsp90 inhibitors for cancer treatment. Pyrimidine analogs interrupt Hsp90 ATP hydrolyses process through disrupting N terminal domain (NTD) conformational change (Koca, İ, et al. *Eur. J. Med. Chem.* **2016**, *122*, 280–290). Coumarin derivative compounds inhibit C terminal domain (CTD) of Hsp90, and block dimerization process (Koca, İ., et al., *Anticancer Agents Med. Chem.* **2015**, *15*, 916–930). As an alternative to Hsp90 inhibitors, Hsp70 substrate binding domain (SBD) inhibitors are designed and synthesized for effective cancer treatment. Our results indicated that these inhibitors provide significant opportunities for cancer treatment.

**Acknowledgments:** The authors gratefully acknowledge the financial support received from the Scientific and Technological Research Council of Turkey, TÜBİTAK (Grant # 114Z365).

### 2.24. Design and In Vitro Testing of New Antimicrobial Peptides Based on QSAR Modelling (A031)

VishnepolskyBoris^1^*GrigolavaMaya^1^ZaalishviliGiorgi^2^KarapetianMargarita^2^PirtskhalavaMalak^1^*1I. Beritashvili Center of Experimental Biomedicine, Gotua str. 14, Tbilisi 0160, Gerogia2Agricultural University of Georgia, 240 David Aghmashenebeli Alley, Tbilisi 0159, Georgia*Correspondence: b.vishnepolsky@lifescience.org.ge (B.V.); m.pirtskhalava@lifescience.org.ge (M.P.)

Antimicrobial peptides (AMPs) are anti-infectives that may represent a novel and untapped class of biotherapeutics. In the lab of bioinformatics of IBCEB, the Database of Antimicrobial Activity and Structure of Peptides (Pirtskhalava, M., et al. *Nucl. Acids Res.* **2016**, *44*, D1104–D1112) has been developed. DBAASP provides the information and analytical resources to the scientific community in order to develop antimicrobial compounds with the high therapeutic index.

Quantitative structure-activity relationship (QSAR) studies for the development of predictive model for AMP are generally based on discriminative analysis and especially machine learning methods. These methods, as a positive training set, have used a full set of antimicrobial peptide sequences, without taking into account variation in mechanisms of action, structure, mode of interaction with membrane and other differences. Contrary to available approaches, we think that strategy of prediction should be based on the fact that there are at least four kinds of AMPs for which four independent algorithms of prediction have to be developed in order to get high efficacy. We can distinguish linear cationic antimicrobial peptides (LCAP), cationic peptides stabilizing structure by intra-chain covalent bonds, proline and arginine-rich peptides, and anionic antimicrobial peptides.

Simple predictive model, which can discriminate AMP from non-AMP has been developed for LCAP. Sequences have been taken from DBAASP. As descriptors, the sequence-based physical-chemical characteristics responsible for capability of the peptide to interact with an anionic membrane were considered. On the basis of these characteristics, a new simple algorithm of prediction is developed and in silico evaluation of the efficacies of characteristics is done. The algorithm was based on the AMP clusterization by their physicochemical properties. The results show that descriptors relied mainly on hydrophobic and hydrophilic features allow us to predict AMP with the high accuracy.

The developed predictive model was used to design new peptides. Antimicrobial potency of these peptides has been evaluated by in vitro testing of peptides’ activity against different bacteria (including drug resistant strains). In vitro estimation shows high accuracy of the developed predictive model.

**Acknowledgments:** This work was supported by International Science and Technology Center provided through National Institute of Allergy and Infectious Diseases/National Institutes of Health (G-2102) and Shota Rustaveli National Science Foundation (FR/397/7-180/14).

### 2.25. Antimicrobial Activity of Various Hydantoin Derivatives (A032)

RadojevićIvana D.ĐukićMarijana V.ČomićLjiljana R.AšaninDarko P.ŠmitBiljana M.*Faculty of Science, University of Kragujevac, Radoja Domanovića 12, 34000 Kragujevac, Serbia*Correspondence: biljam@kg.ac.rs

A series of 22 synthetic drug-like hydantoin derivatives, including aryl or alkenyl 5,5-disubstituted hydantoins, spirohydantoins and annulated bicyclic and tricyclic hydantoins, was assayed for in vitro antibacterial and antifungal activity. The antimicrobial activity was tested by determination of the minimum inhibitory concentration (MIC) and the minimum bactericidal concentration (MBC) using microdilution method. The tested hydantoin derivatives showed moderate antibacterial and weak antifungal activity. The intensity of acting varied depending on the structure and concentration of the test substances and the type of test organisms. The bicyclic benzeneselenenyl derivatives of hydantoin have shown the highest inhibitory activity. The tested compounds appear promising for a fragment-based drug design approach and further bioactivity studies.


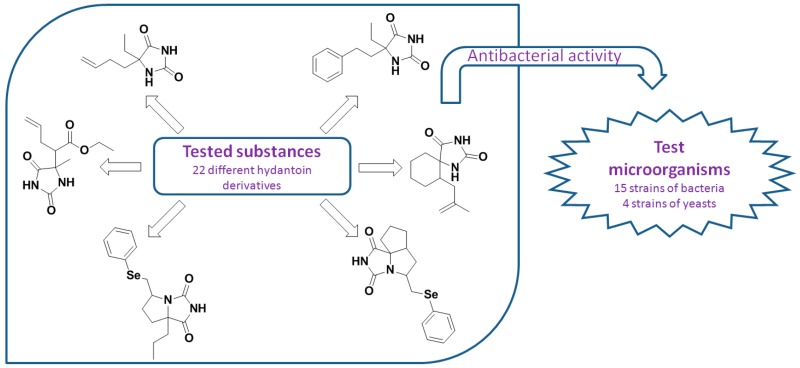


**Acknowledgments:** This work was supported by the Ministry of Education, Science and Technological Development of the Republic of Serbia (Pr. Nos. 172016 and 172047). This research is part of the scientific activity of the international multidisciplinary “SeS Redox and Catalyses” network.

### 2.26. Triazolylpyridazinones as a New Class of Antihypertensive Agents: Design, Synthesis and in vivo Screening (A035)

MishraRavinesh^1^*SiddiquiAnees A.^2^HusainAsif^2^BhardwajSweta^3^RashidMohd^2^1School of Pharmacy and Emerging Sciences, Baddi University of Emerging Sciences and Technology, Baddi (Solan), Himachal Pradesh 173205, India2Department of Pharmaceutical Chemistry, Faculty of Pharmacy, Jamia Hamdard, New Delhi 110062, India3Department of Chemistry, University School of Sciences, Rayat Bahra University, Mohali, Punjab 140104, India*Correspondence: ravikcp@gmail.com

The chemistry of pyridazinones has been an interesting field of study for decades. The synthesis of novel pyridazinone derivatives and investigation of their chemical and biological behavior have gained more importance in recent decades for biological, medicinal, and agricultural reason. Living organisms finds it difficult to construct N–N bonds, which limits the natural abundance of compounds having such bonds. The pharmacological activity of 4,5-dihydro-6-phenyl-3(2*H*)-pyridazinones has been extensively studied and they are known for their cardiovascular effects. In this field several compounds such as zardaverine or imazodan have been developed as PDE III inhibitors in the search for new antiplatelet or cardiotonic agents. A number of 6-(substituted-phenyl)-2-(4-substituted-phenyl-5-thioxo-4,5-dihydro-1*H*-1,2,4-triazol-3-yl)-4,5-dihydropyridazin-3(2*H*)-one derivatives were designed and synthesized by a sequence of reactions starting from the respective aryl hydrocarbons. The final compounds **4a**–**4u** were evaluated for antihypertensive activities by the non-invasive Tail Cuff method. The compounds **4e**, **4i** and **4k** showed appreciable antihypertensive activity comparable with that of standard hydralazine and propranolol.

### 2.27. Phytosterols: A Healthy Alternative to Cholesterol? (A037)

O’ConnellNiamh M.^1^FoleyDavid A.^1^O’CallaghanYvonne^2^KennyOlivia^2^O’BrienNora M.^2^MaguireAnita R.^1^McCarthyFlorence O.^1^*1Department of Chemistry, Analytical and Biological Chemistry Research Facility, University College Cork, Cork, Ireland2School of Food and Nutritional Sciences, University College Cork, Cork, Ireland*Correspondence: f.mccarthy@ucc.ie

Phytosterols are increasingly used as health supplements in functional foods and are associated with having both positive and negative effects on health (Brendsel, J. et al. *Biochem. Biophys. Res. Comm*. **2014**, *446,* 786–791). In contrast to the heavily promoted health benefits of dietary phytosterol supplementation, a number of groups have identified adverse health effects of phytosterols: induction of endothelial dysfunction and increased size of ischaemic stroke; inhibition of cell growth; aggressive vascular disease in sitosterolaemic patients. (Weingartner, O. et al. *J. Am. Coll. Card.* **2008**, *51*, 1553–1561; Ryan, E. et al. *Br. J. Nut*. **2005**, *94*, 443–451). Given this disparity, an investigation of their full individual biological profile is imperative in order to assure food safety.

Herein we describe the de novo synthesis of pure phytosterols on a multigram scale and report the first synthesis of the key phytosterol dihydrobrassicasterol and its oxides along with a comparison of routes to campesterol (McCarthy, F.O. et al. *Org. Biomol. Chem*. **2005**, *3*, 3059–3065; O’Connell, N.M. et al. *Tetrahedron* **2012**, *68*, 4995–5004). A detailed spectroscopic analysis is included, with full assignment of the ^13^C-NMR spectra of both phytosterols, mixtures and their precursors, illustrating to the potential use of NMR as a tool for analysis of these sterol mixtures. A comprehensive toxicological profile of these key phytosterol oxide products (POPs) identifies critical problems with the use of phytosterol mixtures as food additives.5,6,7 (O’Callaghan, Y. et al. *J. Agric. Food Chem*. **2010,** *58*, 0793-10798; O’Callaghan, Y. et al. *Biochimie* **2013**, *95*, 496-503; Foley, D.A. et al. *J. Agric. Food Chem.* **2010**, *58*, 1165–1173; Kenny, O. et al. *J. Agric. Food Chem.* **2012**, *60*, 5952–5961).

**Acknowledgments:** The authors wish to acknowledge that this research was funded under the National Development Plan, through the Food Institutional Research Measure (06RDC427: Phytosterol oxides: a complete individual toxicological profile) administered by the Department of Agriculture, Fisheries and Food, Ireland.

### 2.28. Chiral Derivatives of Xanthones: Investigation of Enantioselectivity as Inhibitors of Cyclooxygenases (COX-1 and COX-2) and Binding Interaction with Human Serum Albumin (A023)

FernandesCarla^1^,^2^PalmeiraAndreia^1^,^2^RamosInês I.^1^KennyCarlos^1^AfonsoCarlos^1^,^2^TiritanM. Elizabeth^1^,^2^,^3^CidadeHonorina^1^,^2^PintoPaula C.A.G.^4^SaraivaM. Lúcia M.F.S.^4^ReisSalette^4^PintoMadalena M.M.^1^,^2^*1Laboratório de Química Orgânica e Farmacêutica, Departamento de Ciências Químicas, Faculdade de Farmácia, Universidade do Porto, Rua Jorge Viterbo Ferreira nº 228, 4050-313 Porto, Portugal2Centro Interdisciplinar de Investigação Marinha e Ambiental (CIIMAR), Universidade do Porto, Edifício do Terminal de Cruzeiros do Porto de Leixões, Av. General Norton de Matos s/n, 4050-208 Matosinhos, Portugal3CESPU, Instituto de Investigação e Formação Avançada em Ciências e Tecnologias da Saúde, Rua Central de Gandra 1317, 4585-116 Gandra PRD, Portugal4REQUIMTE, Departamento de Ciências Químicas, Faculdade de Farmácia, Universidade do Porto, Rua de Jorge Viterbo Ferreira, 228, 4050 313 Porto, Portugal*Correspondence: madalena@ff.up.pt

The search for new enantiomerically-pure chiral derivatives of xanthones (CDXs) with potential pharmacological properties—i.e., with anti-inflammatory activity—has long been an area of interest in our group (Fernandes, C., et al. * Bioorg. Med. Chem.* **2014**, *22*, 1049–1062; Fernandes, C., et al. *Eur. J. Med. Chem.* **2012**, *55, *1–11). Herein, we describe in silico studies and in vitro inhibitory assays of different enantiomeric pairs of CDXs. The evaluation of the inhibition of cyclooxygenases (COX-1 and COX-2) activities was performed by using the COX Inhibitor Screening Assay Kit. Docking simulations between the small molecules (CDXs, known ligands and decoys) and the enzyme targets were undertaken with AutoDock Vina embedded in PyRx—Virtual Screening Tool software. As predicted, all the CDXs evaluated exhibited COX-1 and COX-2 inhibition potential.

As the (*S*)-(−)-enantiomer of the nonsteroidal anti-inflammatory drug, Ketoprofen, preferentially binds to albumin, resulting in lower free plasma concentration than (R)-(+)-enantiomer (Evans, S.E., et al. *Trends Environ. Anal. Chem.* **2014**, *1*, e34–e51), protein binding affinity for CDXs was also evaluated by spectrofluorimetry. For some CDXs enantioselectivity was observed.

**Acknowledgments:**This research was partially supported by the Structured Program of R&D&I INNOVMAR—Innovation and Sustainability in the Management and Exploitation of Marine Resources (reference NORTE-01-0145-FEDER-000035, Research Line NOVELMAR), funded by the Northern Regional Operational Programme (NORTE2020) through the European Regional Development Fund (ERDF), and by the Foundation for Science and Technology (FCT) and COMPETE under the projects PTDC/MAR-BIO/4694/2014 (POCI-01-0145-FEDER-016790) and COXANT–CESPU-2016.

## 3. Conclusions

The Second International Electronic Conference on Medicinal Chemistry gathered more than 150 authors from twenty-two countries (Austria, Belgium, Brazil, France, Georgia, Germany, India, Ireland, Malta, Pakistan, Portugal, Romania, Russia, Serbia, Slovakia, Spain, Switzerland, Taiwan, Turkey, Ukraine, USA, and Venezuela). It attracted 14 media partners which had the opportunity to inaugurate our new virtual exhibition hall. The best presentation, as elected by the Scientific Committee of the Conference, was the work entitled “Design, Synthesis and Biological Activity of Furoxan Derivatives against Multi-drug Resistant Tuberculosis” (A026) submitted by G. Fernandes, P. Souza, C.M. Chin, F. Pavan, and J.L. Dos Santos, from the University Estadual Paulista at Araraquara, Brazil. It has rewarded by a special travel grant offered by our Journal.

Thanks to the support of the organizers and the confidence of the participants, we are proud to announce that the Third International Conference on Medicinal Chemistry will be held in November 2017 on www.sciforum.net/conference/ecmc-3. All of us hope that you will have the opportunity to attend, as authors or visitors.

## Supplementary Materials

The full presentations are available online at www.sciforum.net/conference/ecmc-2.

